# Frequency of Medical Claims for Diastasis Recti Abdominis Among U.S. Active Duty Service Women, 2016 to 2019

**DOI:** 10.1089/whr.2023.0012

**Published:** 2023-10-09

**Authors:** Jessica Korona-Bailey, Amanda Banaag, Penelope Jones, Dana R. Nguyen, Tracey Pérez Koehlmoos

**Affiliations:** ^1^ Uniformed Services University of the Health Sciences, Bethesda, Maryland, USA.; ^2^ Henry M. Jackson Foundation for the Advancement of Military Medicine, Bethesda, Maryland, USA.

**Keywords:** diastasis recti, Military Health System, active duty service women, women's health

## Abstract

**Background:**

Diastasis recti abdominis (DRA) is a condition in pregnant and postpartum women. Proposed risk factors include age, sex, multiparity, cesarean delivery, diabetes, gestational weight gain, and high birth weight. This study aims to estimate the prevalence of DRA using medical claims data among U.S. active duty service women (ADSW) and determine associated risk factors.

**Materials and Methods:**

We conducted a cross-sectional study of ADSW aged 18 years and older in the U.S. Army, Air Force, Navy, and Marine Corps during fiscal years (FYs) 2016 to 2019. Utilizing claims data, we identified ADSW with a diagnosis of DRA during the study period. Risk factors, including age, race, socioeconomic status, branch of service, military occupation, delivery type, and parity, were evaluated through descriptive statistics, chi-square tests, and logistic regression analysis.

**Results:**

A total of 340,748 ADSW were identified during FYs 2016 to 2019, of whom 2,768 (0.81%) had a medical claim for DRA. Of those with deliveries during the study period, 1.41% were multiparous and 84.53% had a cesarean delivery. Increased risk of DRA was found in ages 30 to 39 years, Black women, ranks representing a higher socioeconomic status, and women with overweight and obese body mass indices.

**Conclusions:**

Although the prevalence of DRA, defined as a medical claim for DRA, in the study population is low, subpopulations may be disproportionately affected by the condition. Further research could potentially detail the impact of DRA on the functional impairment and operational readiness of ADSW in the U.S. military and any possible means of prevention.

## Introduction

Diastasis recti abdominis (DRA) is an acquired condition defined as the midline separation of the rectus abdominis muscle due to widening or weakening of the linea alba.^1–4^ The condition is common in pregnant and postpartum women; however, due to differences in assessment methods, prevalence ranges vary widely from 27% to 100% during pregnancy and 30% to 68% in the postpartum period.^1–4^ Additionally, DRA is likely underdiagnosed, as documented by health care providers, due to low patient reporting or lack of provider awareness or understanding of the condition. As a result, most published studies on DRA have small sample sizes.^1,3^

To date, there is no consensus on the risk factors for developing DRA; however, proposed and researched factors include older age, sex, multiparity, cesarean delivery, diabetes, gestational weight gain, and high birth weight.^1,2,4,5^ Furthermore, one study reported an increased likelihood of DRA among women exposed to frequent heavy weight lifting.^5^

While some believe that diastasis recti is a cosmetic concern, the condition may contribute to reduced trunk stability, low back or lumbo-pelvic pain, abdominal pain or weakness, and urinary incontinence.^1–4^ Treatment options for DRA include both surgical and nonsurgical methods, but there is a lack of clear clinical practice guidelines for best practices for either method.^6^ Despite the lack of established best practices, treatment often begins with physical therapy and may escalate to surgical repair if insufficient improvement is made.

Literature and research studies focusing on women and pregnancy-related conditions are lacking. An often overlooked population for research is the Military Health System (MHS), which serves over 1 million active duty service women (ADSW), including women at risk for DRA.^7^ The MHS is a bifurcated system that provides its beneficiaries, including active duty service members, retirees, and their dependents, access to care in two ways: through direct care from providers at military treatment facilities or through private sector care, from civilian fee-for-service facilities that accept the TRICARE insurance benefit.^8^

Moreover, ADSW constitute 17% of the U.S. military force^7^ and many serve in military occupations with physical requirements of heavy lifting and/or the operation of heavy machinery.^8^ All service members must also pass periodic physical fitness tests that assess cardiovascular endurance, muscular strength, and functional mobility—many components of which necessitate abdominal strength. DRA is of particular concern for ADSW given these occupational and physical fitness requirements.

For postpartum ADSW in particular, the presence of DRA can impair their ability to meet physical fitness requirements and successfully return to duty. Despite the potential impact on readiness, aside from a case study, little is known about DRA in ADSW.^9^ The aim of this study is to determine the prevalence of DRA among ADSW and associated risk factors using medical claims data.

## Materials and Methods

### Data source and study design

We used the MHS Data Repository (MDR) to conduct a cross-sectional study of ADSW in the U.S. Army, Air Force, Navy, and Marine Corps during fiscal years (FYs) 2016 to 2019. The MDR houses administrative and health care claims data for MHS beneficiaries, including active duty service members, retirees, and their dependents; however, claims data do not capture care delivered in combat zones or through the Veterans Health Administration.^10^ Data from the MDR have been used in previous studies investigating the health of ADSW.^8,11^

### Study population

Using the Defense Enrollment Eligibility Reporting System (DEERS) in the MDR, we identified all ADSW aged 18 years and older from FY 2016 to 2019. Women in the National Guard or Reserves, both active and inactive, were excluded due to their inconsistent access to care in the MHS. Utilizing International Classification of Diseases, 10th Revision (ICD-10) codes, Medicare Severity Diagnosis-Related Group (MS-DRG) codes, and Current Procedure Terminology (CPT) codes, we identified ADSW with a diagnosis of DRA, with and without pregnancy, and concurrent treatments or repair procedures.

A full list of the ICD-10 and CPT codes used can be found in Supplementary Appendix Table SA1. The earliest record within the study period was retained.

### Grouping variables (risk factors and characteristics)

Risk factors for DRA, including age, parity, mode of delivery, and body mass index (BMI), were identified for ADSW in the study population. BMI classification was determined using the following standard categorization: underweight (<18.5 kg/m^2^), healthy weight (18.5–24.9 kg/m^2^), overweight (25–29.9 kg/m^2^), and obese (≥30 kg/m^2^). For those with DRA, BMI was calculated for the FY of diagnosis, and for those without DRA, BMI was calculated for the earliest FY of their DEERS record during the study period.

Mode of delivery was identified using ICD-10 and MS-DRG codes in Supplementary Appendix Table SA1. Parity was determined by identifying all deliveries per person during the study period that were at least 40 weeks apart and preceded the date of DRA diagnosis. For those women who received a DRA diagnosis at the beginning of the study period (FY 2016), records from FY 2015 were reviewed for deliveries that occurred in the year preceding the diagnosis.

Physical therapy, reevaluation physical therapy, and surgical repair were identified and coded as binary variables (0 = no and 1 = yes) and marked as “yes” if the patient received therapy at any time after the diagnosis of DRA. The combination of treatment received or the hierarchy of occurrence was not evaluated in this study.

Patient demographics, such as race, branch of service, rank, BMI, and military occupational specialty (MOS), were obtained from the health care claim at the time of DRA diagnosis or, for those without DRA, from the earliest DEERS record within the study period.

### Statistical analyses

Descriptive statistics were used to analyze patient demographics and military service-related characteristics (age group, race, military service rank, branch of service, MOS, and BMI category) for the total population and by DRA diagnosis. The prevalence of DRA in ADSW was calculated and expressed as a percentage. Group differences between ADSW with and without DRA diagnosis were analyzed utilizing the chi-square test for independence. Univariate logistic regression analysis was performed on each categorical variable to assess its association with DRA diagnosis in ADSW.

To control for confounding factors, a subsequent multivariable logistic regression was performed and adjusted by all six predictive factors. Any observations with missing values were automatically removed from the logistic regression analyses. For all analyses, *p*-values <0.05 were considered statistically significant, and analyses were conducted using SAS, version 9.4. The study was considered exempt by the Institutional Review Board of the Uniformed Services University of the Health Sciences.

## Results

We identified a total of 340,748 ADSW from the MDR in FYs 2016 to 2019, of whom 2,768 (0.81%) had a medical claim for DRA during the study period. Descriptive demographic data for the total study population of ADSW and ADSW with DRA during the study period are presented in Table 1. Due to a very small number of ADSW being classified as underweight, this category of BMI was censored from the descriptive statistics. The majority of ADSW in the total study population were between 20 and 29 years of age (51.62%), White (57.46%), in a junior enlisted rank (62.02%), and categorized as being of healthy weight (39.59%).

**Table 1.** Demographics of Active Duty Service Women, Fiscal Years 2016–2019

	Total ADSW (***N*** = 340,748)	ADSW w/diastasis recti (***N*** = 2,768) ***n*** (column %)	Row %	Chi-square ***p***-value
Age group, years				
18 to 19	86,732 (25.66)	65 (2.35)	0.07	<0.0001
20 to 29	174,483 (51.63)	1,424 (51.45)	0.81	
30 to 39	58,202 (17.22)	1,125 (40.64)	1.90	
40 and older	18,563 (5.49)	154 (5.56)	0.82	
Race				
White	194,438 (57.53)	1,368 (49.42)	0.70	<0.0001
Black	90,147 (26.67)	1,050 (37.93)	1.15	
Asian/Pacific Islander	22,745 (6.73)	152 (5.49)	0.66	
American Indian/Alaska Native	4,550 (1.35)	36 (1.30)	0.78	
Other	14,980 (4.43)	115 (4.15)	0.76	
Missing	11,120 (3.29)	47 (1.70)	0.42	
Rank				
Junior enlisted	210,396 (62.25)	947 (34.21)	0.45	<0.0001
Senior enlisted	72,579 (21.47)	1,194 (43.14)	1.62	
Junior officer	42,135 (12.47)	551 (19.91)	1.29	
Senior officer	4,911 (1.45)	50 (1.81)	1.01	
Warrant officer	1,705 (0.50)	26 (0.94)	1.50	
Other	6,254 (1.85)	0	0	
Service branch				
Army	118,559 (35.08)	1,253 (45.27)	1.05	<0.0001
Air Force	93,229 (27.58)	869 (31.39)	0.92	
Navy	99,209 (29.35)	536 (19.36)	0.54	
Marine Corps	26,983 (7.98)	110 (3.97)	0.41	
Military occupation^a^				
Administration/support	61.187 (18.10)	661 (23.88)	1.07	<0.0001
Aviation	15,823 (4.68)	88 (3.18)	0.55	
Communications/intelligence	30,975 (9.16)	311 (11.24)	0.99	
Engineering/repair/maintenance	20,262 (6.00)	429 (15.50)	2.07	
Health care	48,305 (14.29)	556 (20.09)	1.14	
Law enforcement/security	11,184 (3.31)	79 (2.85)	0.70	
Motor transport	11,170 (3.30)	102 (3.68)	0.90	
Naval transport	11,683 (3.46)	53 (1.91)	0.45	
Supply/logistics	19,114 (5.66)	168 (6.07)	0.87	
Warfighter/combat specialist	9,287 (2.75)	70 (2.53)	0.75	
Other	79,814 (23.62)	247 (8.92)	0.31	
BMI category				
Unknown	68,711 (20.33)	52 (1.88)	0.08	<0.0001
Underweight	^b^	^b^	^b^	
Healthy weight	133,998 (39.65)	915 (33.06)	0.68	
Overweight	102,274 (30.26)	1,149 (41.51)	1.11	
Obese	30,317 (8.97)	642 (23.19)	2.07	

^a^ Missing occupation excluded from the table.

^b^ Censored due to small cell size and to prevent back calculation.

ADSW, active duty service women; BMI, body mass index.

By branch of service, a greater proportion of the total study population were in the Army (35.16%), followed by Navy (29.27%), Air Force (27.62%), and Marine Corps (7.95%). Occupationally, the largest group was a composite group, “Other” (23.50%), followed by “Administration/support” (18.15%) and “Health care” (14.34%). Due to the large proportion of “Other” occupations, we have included a stratified table of occupations categorized as “Other” in Supplementary Appendix Table SA2.

DRA also appears to disproportionally affect Black ADSW, composing ∼26% of the total study population, but carrying ∼38% of DRA diagnoses. Likewise the frequency of claims for DRA in Black ADSW was 1%, more than the proportion experienced by each of the other racial groups. Similarly, those classified as obese compose over 23% of DRA diagnoses, but make up ∼9% of the total study population.

However, the condition was most frequent in ADSW who were overweight (41.51%), followed by those with healthy weight (33.06%). The frequency of claims in obese ADSW was 2% compared with 1% for overweight ADSW. Among ADSW with a medical claim for DRA, ∼63% received physical therapy or were referred to a physical therapist for reevaluation and just over 8% underwent a surgical intervention.

**FIG. 1.**
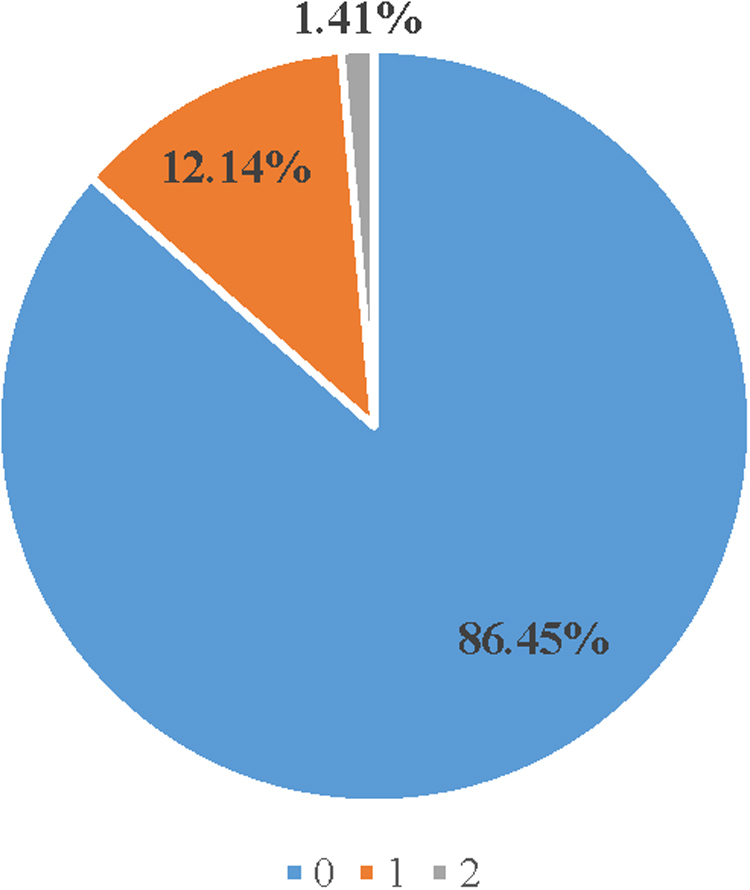
Number of deliveries among ADSW with DRA. ADSW, active duty service women; DRA, diastasis recti abdominis.

**FIG. 2.**
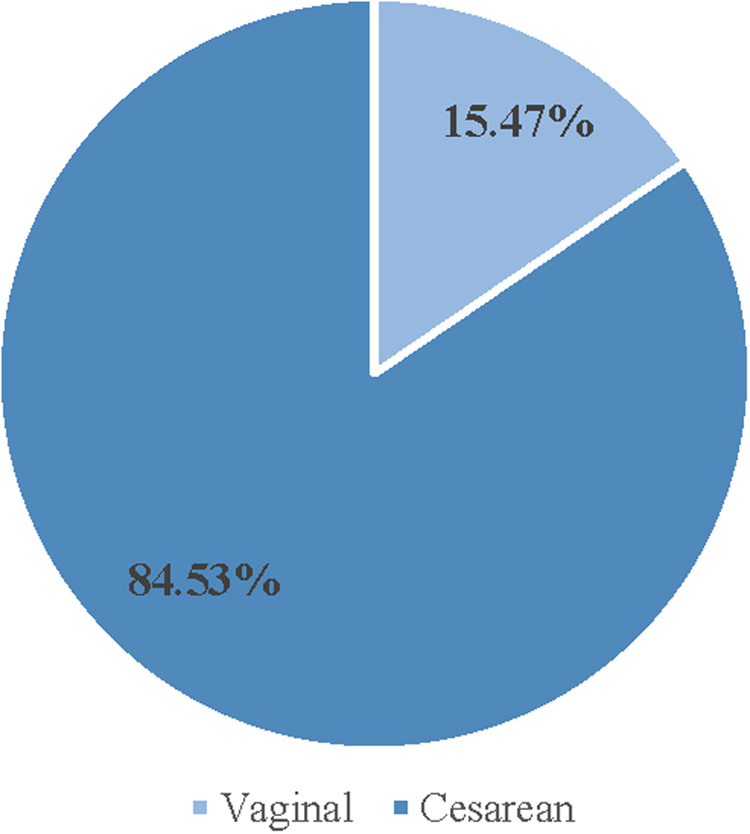
Mode of delivery among ADSW with DRA.

For ADSW with DRA, we identified whether or not they had given birth during the study period and the mode of delivery. The majority had not given birth during the study period nor in the year immediately preceding their diagnosis (none, *n* = 2,393 [86.45%]; delivered once, *n* = 336 [12.14%]; and delivered twice, *n* = 39 [1.41%]) (Fig. 1). Of those who gave birth, the majority had a cesarean delivery (*n* = 317 [84.53%]) (Fig. 2).

Table 2 illustrates the unadjusted and adjusted logistic regression results for the odds of DRA in ADSW during the study period. From the adjusted results and compared with their referent groups, we observed higher odds of DRA in ADSW between ages 30 and 39 years (adjusted odds ratio [aOR] 1.42; confidence interval [95% CI] 1.29–1.56); who are Black (aOR 1.37; 95% CI 1.26–1.50); with a rank above junior enlisted (the highest odds observed in senior officers) (aOR 2.30; 95% CI 1.66–3.20); who have an obese or overweight BMI (aOR 2.14; 95% CI 1.92–2.38; aOR 1.37; 95% CI 1.25–1.50, respectively); and in occupational categories of Engineering/maintenance/repair (aOR 2.91; 95% CI 2.56–3.31) and Other (aOR 1.33; 95% CI 1.13–1.57).

**Table 2.** Unadjusted and Adjusted Logistic Regression Results for the Odds of Diastasis Recti in Active Duty Service Women, Fiscal Years 2016–2019

	Unadjusted OR (95% CI)	Adjusted OR (95% CI)
Age group, years		
18 to 20	0.09 (0.07–0.12)^a^	0.16 (0.12–0.21)^a^
20 to 29 (reference)	1	1
30 to 39	2.37 (2.19–2.56)^a^	1.42 (1.29–1.56)^a^
40 and older	1.02 (0.86–1.20)	0.52 (0.42–0.63)^a^
Race		
White (reference)	1	1
Black	1.66 (1.53–1.80)^a^	1.37 (1.26–1.50)^a^
Asian/Pacific Islander	0.95 (0.80–1.12)	0.77 (0.65–0.91)^a^
American Indian/Alaska Native	1.13 (0.81–1.57)	1.03 (0.74–1.44)
Other	1.09 (0.90–1.32)	1.05 (0.86–1.27)
Rank		
Junior enlisted (reference)	1	1
Senior enlisted	3.66 (3.36–3.98)^a^	1.79 (1.61–2.00)^a^
Junior officer	2.91 (2.61–3.23)^a^	1.83 (1.62–2.07)^a^
Senior officer	2.26 (1.70–3.01)^a^	2.30 (1.66–3.20)^a^
Warrant officer	3.39 (2.29–5.02)^a^	1.50 (1.00–2.25)^a^
Service		
Army (reference)	1	1
Air Force	0.88 (0.81–0.96)^a^	0.77 (0.70–0.85)^a^
Navy	0.39 (0.32–0.47)^a^	0.49 (0.38–0.49)^a^
Marine Corps	0.51 (0.46–0.57)^a^	0.59 (0.48–0.72)^a^
BMI category		
Underweight	0.55 (0.29–1.02)	0.56 (0.29–1.08)
Healthy weight (reference)	1	1
Overweight	1.65 (1.51–1.80)^a^	1.37 (1.25–1.50)^a^
Obese	3.10 (2.80–3.43)^a^	2.14 (1.92–2.38)^a^
Unknown	0.11 (0.08–0.15)^a^	0.16 (0.12–0.22)^a^
Military occupation		
Administration/support (reference)	1	1
Aviation	0.52 (0.41–0.64)^a^	0.79 (0.63–1.00)
Communications/intelligence	0.93 (0.81–1.06)	1.10 (0.96–1.27)
Engineering/repair/maintenance	1.96 (1.73–2.22)^a^	2.91 (2.56–3.31)^a^
Health care	1.07 (0.95–1.19)	1.12 (0.99–1.26)
Law enforcement/security	0.65 (0.52–0.83)^a^	0.84 (0.66–1.06)
Motor transport	0.85 (0.69–1.04)	1.02 (0.82–1.26)
Naval transport	0.42 (0.32–0.56)^a^	1.04 (0.78–1.40)
Supply/logistics	0.81 (0.69–0.96)^a^	0.94 (0.79–1.12)
Warfighter/combat specialist	0.70 (0.55–0.89)^a^	1.05 (0.82–1.36)
Other	0.29 (0.25–0.33)^a^	1.33 (1.13–1.57)^a^

^a^ Statistically significant with *p*-value <0.05.

CI, confidence interval; OR, odds ratio.

Those with fewer medical claims for DRA when compared with their referent group included ADSW aged 18 to 20 years and 40 years or older (aOR 0.16, 95% CI 0.13–0.21, and aOR 0.52, 95% CI 0.42–0.63, respectively), who are Asian/Pacific Islanders (aOR 0.77; 95% CI 0.65–0.91), and in all other branches of services and those in the Navy with the highest protective odds (aOR 0.49; 95% CI 0.43–0.54). Similar results were observed in the unadjusted regression analyses, excluding the unadjusted analyses of military occupation.

## Discussion

We identified 340,748 ADSW, of whom 2,768 had a medical claim for DRA from FY 2016 to 2019. Overall, prevalence was <1% and of those diagnosed through claims data, the majority received the standard treatment of physical therapy. In consideration of findings from both the unadjusted and adjusted logistic regression analyses, all six proposed risk factors contained statistically significant risks of DRA to ADSW in the study population. Of these, age, race, service, rank, and BMI significantly increase the odds of ADSW being diagnosed with DRA.

Although the prevalence of DRA in this study population was low (0.81%) and estimated to be significantly lower than adult women in the general population (27%–100% during pregnancy and 30%–68% in the postpartum period),^1,4^ our findings suggest that subpopulations of ADSW may be disproportionately affected by the condition and warrant further study.

For example, ADSW under the age of 20 or over the age of 40 were least likely to suffer from the condition, which may be due, in part, to their reduced likelihood of being either pregnant or postpartum when compared with women between 20 and 29 years of age. Conversely, ADSW between the ages of 30 and 39 years were disproportionately affected by DRA. This corresponds with literature showing higher prevalence of DRA in women 20 to 40 years of age regardless of the mode of delivery.^12^

Additional risk factors for DRA, such as cesarean delivery and parity, were examined in this study and results showed that 11% of ADSW with DRA underwent a cesarean section and 12% had at least one pregnancy during the study period or in the year prior. These percentages are low given evidence in the literature suggesting that both cesarean deliveries^1^ and parity increase the risk of DRA.^1,4,5^

Additional subpopulations of ADSW appear disproportionately affected by DRA. Black ADSW compose roughly 27% of the total population, but bear nearly 38% of DRA diagnoses. Racial disparity results from this study differ from past studies that show a higher percentage of DRA in White or Asian women.^13^

Many racial disparities in health outcomes are mitigated in the MHS^14^; however, as this study demonstrates, inequities still exist. Overall, very few studies assess race when determining the prevalence of DRA, which is concerning given the growing body of evidence showing racial disparities in women's health.^15,16^

As with previous studies using MHS data, rank is used as a proxy for socioeconomic status when analyzing MHS data.^17,18^ In this analysis, senior officers carried greater risk of DRA. This result is interesting given the existing evidence that enlisted service members traditionally experience worse health outcomes overall compared with service members of officer rank.^19,20^

Additionally, it can be speculated that junior enlisted women may have less structural access to medical care or that their complaints are not being documented. Alternatively, a higher prevalence in senior officers may be associated with age-related risk described above.

Aside from the senior officer rank, the other factor showing the greatest odds for DRA in this study was BMI. Overweight and obese ADSW had greater odds of DRA compared with those with normal weight. This is in accordance with risk factors identified in other studies but conflicts with other studies showing no increased risk with BMI.^4,21,22^ The high percentage of overweight and obese ADSW is concerning given readiness standards; however, it is not surprising.

Numerous studies over the years have cited increasing rates of overweight and obesity in the armed forces.^23,24^ While some weight gain in this population may be attributed to pregnancy, most women return to their previous BMI category postpartum, suggesting that the weight before pregnancy may be an important factor to focus on in terms of prevention.^25^

Treatment in this study appears appropriate with recommendations. Treatment of DRA ranges from conservative approaches (core strengthening exercises) to surgical repair, with most providers favoring nonsurgical treatment. In our population, 63% of women with a diagnosis of DRA had an associated referral or visit for physical therapy. This finding is promising and indicates by proxy that the majority of women who were seen within the health care system received the standard of care treatment.

For those ADSW not seen within the health care system, it is important to note that over the last two decades, Services have implemented evidence-based, comprehensive, postpartum physical training plans aimed at proactively addressing the physical needs of postpartum women.^26^ It is possible that postpartum patients might alternatively enroll into a military-specific program.

While studies have evaluated the individual effectiveness of military programs, there is a need to comprehensively evaluate utilization and long-term functional outcomes between different arms of care (usual care vs. health care system vs. military physical therapy and training programs).

Our study evaluates DRA diagnosis through medical claims data among ADSW, a universally insured population with no financial barriers in access to care. The finding of <1% of DRA cases in our population compared with estimated general population rates is unanticipated. One author's clinical experience with pregnant ADSW suggests that postpartum physical ailments such as DRA and urinary incontinence are widespread and undertreated.

We hypothesize that the low prevalence rate in our study is due, in part, to electronic health record underreporting/undercoding or underdiagnosis by health care providers. Both of these factors could lead to low prevalence, as defined by ICD-10 coding, and echo other studies describing difficulties defining and studying this condition.^1,3^ While musculoskeletal (MSK) assessments and rehabilitation plans are critical to returning the service member to full duty, providers often lack confidence and/or training in MSK medicine or women's health. Additionally, these services may be lacking or hard to access, as shown in the The Women's Reproductive Health Survey of Active-Duty Service Members.^27^

Another consideration is that ADSW have fitness requirements that include a focus on core or abdominal strength measured by performance of the plank among others. These ADSW could be considered stronger than the average civilian and possibly tougher, not voicing their complaints, further leading to the underreporting and underdiagnosis of DRA among ADSW.

Secondary analyses could compare DRA medical claims between ADSW and female dependents in the MHS to determine if there are differences between the two populations. Many factors come into play impacting DRA among ADSW, and our study can help guide future research as subpopulations at an increased risk for DRA among ADSW are identified.

Future qualitative research would allow us to more fully understand whether the prevalence of DRA is actually lower among ADSW or whether the condition is being underreported.^1,3^

### Limitations

This study had several limitations. First, the diagnosis of DRA was limited to women reporting their condition to a medical provider and the provider concurrently coding the condition correctly. We are most likely underreporting the true prevalence in our study population. Additionally, the use of claims data has the potential for coding errors and inadequate specificity for a condition, and as such, we are unable to describe factors impacting prevalence, as determined by claims data.

Second, this study does not capture data for any health care received outside of the TRICARE benefit. Ascertainment of prior pregnancy was limited to 1 year before DRA diagnosis and pregnancies outside of this window were not included. Although this study identified ADSW with a DRA diagnosis and any concurrent treatments or repair interventions, the study failed to include any related information pertaining to the basis of the clinical visit, such as complaints of pain or discomfort, loss of function, or issues with mobility.

Given the lack of consensus on the level of functional impairment, which can be attributed to DRA, more information is needed to understand the basis behind the complaint, diagnosis, subsequent interventions, and clinical outcomes.

## Conclusions

This study using medical claims data lays the foundation for DRA research in ADSW. Our findings show that although the medical claims of DRA in the total population of ADSW are low, subpopulations may be disproportionately affected by the condition. Future research should include investigations into the disparities between subpopulations of ADSW at greater risk of developing DRA, which could provide information about the impact of DRA on functional impairment and operational readiness and possible means of prevention.

## Supplementary Material

Supplemental data

Supplemental data

## Data Availability

The data that support the findings of this study are available from the U.S. Defense Health Agency. Restrictions apply to the availability of these data, which were used under federal Data User Agreements for the current study and so are not publicly available.

## Disclaimer

The contents of this publication are the sole responsibility of the authors and do not necessarily reflect the views, assertions, opinions, or policies of the Uniformed Services University of the Health Sciences (USUHS), the Henry M. Jackson Foundation for the Advancement of Military Medicine, Inc., (HJF), the Department of Defense (DoD), or the Departments of the Army, Navy, and Air Force. Mention of trade names, commercial products, or organizations does not imply endorsement by the U.S. Government.

## Supplementary Material

Supplementary Appendix Table SA1

Supplementary Appendix Table SA2

## Abbreviations Used

ADSW: active duty service women
BMI: body mass index
CPT: Current Procedure Terminology
DEERS: Defense Enrollment Eligibility Reporting System
DRA: diastasis recti abdominis
FY: fiscal year
ICD-10: International Classification of Diseases, 10th Revision
MDR: MHS Data Repository
MHS: Military Health System
MOS: military occupational specialty
MS-DRG: Medicare Severity Diagnosis-Related Group
MSK: musculoskeletal
