# Supplementary material for: Frequency of Medical Claims for Diastasis Recti Abdominis Among U.S. Active Duty Service Women, 2016 to 2019
Source: Womens Health Rep (New Rochelle). 2023 Oct 9;4(1):470–7. doi: 10.1089/whr.2023.0012 (PMC10561740; doi:10.1089/whr.2023.0012)
Supplement: Supplemental data [file Suppl_AppendixTableSA1.docx]

**Appendix Table 1:** ICD-10, CPT, and MS-DRG codes utilized in the study

| **Diagnosis or Procedure** | **Type of Code** | **Codes** |
| --- | --- | --- |
| DRA | ICD-10 Dx | M62.08, O71.89 |
| Physical Therapy Evaluation | CPT | 97161-97163 |
| Physical Therapy Re-evaluation | CPT | 97164 |
| Surgical | CPT | 15830, 15847 |
| **Delivery** |  |  |
| Delivery | ICD-10 Dx; MS-DRG | O80-O84; 762-768, 771-773, 783-785, 805-807 |
| Vaginal | ICD-10 Dx; MS-DRG | O80; 762-764, 767, 768, 805-807 |
| Cesarean | ICD-10 Dx; MS-DRG | O82; 765, 766, 771-773, 783-785 |
| **Adult BMI** | ICD-10 Dx | Z68.1-Z68.4 |
