# Supplementary material for: Frequency of Medical Claims for Diastasis Recti Abdominis Among U.S. Active Duty Service Women, 2016 to 2019
Source: Womens Health Rep (New Rochelle). 2023 Oct 9;4(1):470–7. doi: 10.1089/whr.2023.0012 (PMC10561740; doi:10.1089/whr.2023.0012)
Supplement: Supplemental data [file Suppl_AppendixTableSA2.docx]

**Appendix Table 2.** Breakdown of Occupations Classified as Other for ADSW, n=80,061

| **Occupation Description** | **Percent of N** |
| --- | --- |
| Not Occupationally Qualified, General | 83.70 |
| Unmatched | 8.20 |
| Students | 3.58 |
| Cadets and Other Officer Candidates | 1.18 |
| Musicians, General | 0.98 |
| Weather, General | 0.91 |
| Non-Occupational, Other | 0.53 |
| Memorial Activities & Embalming | 0.36 |
| Undesignated Occupations, General | 0.28 |
| Physical Science Laboratory | 0.17 |
| Educators & Instructors | 0.08 |
| Other Technical Specialists & Assistants | 0.03 |
| Auxiliaries | <0.03 |
| Chemical | <0.03 |
| Precision Equipment, General | <0.03 |
| Training Administrators | <0.03 |
| Training Devices | <0.03 |
| Not occupationally qualified could be people in training pipelines to include officer candidate school, flight school, or entry level training among others. | |
